# Immunopsychiatry: important facts

**DOI:** 10.1017/S0033291717000745

**Published:** 2017-04-18

**Authors:** G. M. Khandaker, R. Dantzer, P. B. Jones

**Affiliations:** 1 Department of Psychiatry, University of Cambridge, Cambridge, UK; 2 Cambridgeshire and Peterborough NHS Foundation Trust, Cambridge, UK; 3 Department of Symptom Research, University of Texas MD Anderson Cancer Centre, Houston, TX, USA

**Keywords:** Autoimmunity, autoantibody, cytokine, depression, immune system, immunopsychiatry, inflammation, mental disorders, psychiatry, schizophrenia

## Abstract

Accumulating evidence indicate a role for the immune system particularly inflammation and autoimmunity in the aetiology of major psychiatric disorders such as depression and schizophrenia. In this paper, we discuss some of the key advances in immunopsychiatry in order to highlight to psychiatrists and other health professionals how an increased understanding of this field might enhance our knowledge of illness mechanism and approaches to treatment. We present a brief overview of clinical research that link inflammation and autoimmunity with depression and psychosis, including potential role of inflammation in treatment response, current evidence for the effectiveness of immune-modulating treatment for depression and psychosis, and possible role of inflammation in common physical comorbidities for these disorders such as coronary heart disease and diabetes mellitus. Gaining a better understanding of the role of immune system could be paradigm changing for psychiatry. We need collaborations between clinicians and scientists to deliver high-quality translational research in order to fully realise the clinical potential of this exciting and rapidly expanding field.

## Introduction

There are intricate interactions between the immune system and the brain that may offer new mechanistic understanding and insights for novel therapies for psychiatric disorders. In particular, proinflammatory cytokines and circulating autoantibodies can influence the brain leading to changes in mood, cognition and behaviour; so may be relevant for the pathogenesis and treatment of depression, psychosis and other major psychiatric disorders (Raison *et al.* 2006; Dantzer *et al*. 2008; Khandaker *et al.* 2015). In this paper, we present a brief overview of clinical research that link inflammation and autoimmunity with depression and psychosis. We discuss potential role of inflammation in treatment response, current evidence for the effectiveness of immune-modulating treatment for depression and psychosis, and possible role of inflammation in common physical comorbidities for these disorders. Our aim is to inform psychiatrists and other health professionals about a few key advances (see Textbox) in the field of immunopsychiatry that could impact on clinical practice rather than providing a comprehensive literature review regarding the immune system and mental disorders. Unless specified otherwise, in this context inflammation refers to chronic, low-grade systemic inflammation as reflected by increased concentrations of circulating inflammatory markers in peripheral blood such as cytokines and acute phase proteins, rather than inflammation in the classical sense, which is clinically manifest by the so-called cardinal signs.

## Evidence for a role of inflammation in depression

Depression is common in people with a chronic inflammatory illness such as rheumatoid arthritis (Dickens & Creed, 2001). About 30–50% of patients with hepatitis C virus develop depression following treatment with interferon (Bonaccorso *et al.* 2002), which is a potent inducer of inflammatory cytokines. These clinical observations provide clues that inflammation may play a role in depression. Indeed, inducing inflammation in healthy volunteers in experimental setting with a typhoid vaccine leads to depressive symptoms and reduced cognitive performance, which is mediated by increase in circulating interleukin 6 (IL-6) levels (Harrison *et al.* 2009), a pleiotropic, proinflammatory cytokine. Furthermore, systematic reviews and meta-analyses of cross-sectional studies have confirmed that the levels of circulating proinflammatory cytokines such as IL-6, IL-1*β*, tumour necrosis factor alpha (TNF-*α*) and acute phase proteins such as C-reactive protein (CRP) are elevated in acutely depressed patients, which are largely normalised after recovery (Howren *et al.* 2009; Haapakoski *et al.* 2015; Goldsmith *et al.* 2016). However, a limitation of the cross-sectional studies is that they cannot determine whether increase in inflammatory markers is a cause or consequence of illness (i.e. reverse causality).

Recently, longitudinal studies have shown that elevated cytokine levels precede, and so could potentially cause, depressive symptoms. Based on over 4000 participants from the Avon Longitudinal Study of Parents and Children (ALSPAC) birth cohort, we have shown that elevated serum levels of IL-6 in childhood increase risks of developing depressive and psychotic symptoms subsequently in adulthood in a linear, dose–response fashion (Khandaker *et al.* 2014*a*). In this study, evidence for an association persisted after taking into account effects of sociodemographic factors, maternal depression and other potential confounders. A longitudinal association between circulating IL-6 and CRP levels and subsequent development and persistence of depressive symptoms have been reported from other cohorts (Gimeno *et al.* 2009; Zalli *et al.* 2016). Taken together the current evidence indicates that inflammation is unlikely to be merely a consequence of depression or stress (which can cause immune-activation). Inflammation precedes depression so could be a causal risk factor for the illness.

However, observed association between inflammatory markers and depression could still be due to residual confounding, so more definitive studies, such as Mendelian randomization and randomised controlled trials (RCTs), are needed to establish whether the association between inflammation and depression is causal. Elevated CRP levels are associated with somatic (e.g. fatigue, anorexia, altered sleep) rather than psychological symptoms of depression in general population (Jokela *et al.* 2016) and in cancer patients who develop depressive symptoms after interferon treatment (Capuron *et al.* 2002). Therefore, focusing on specific symptoms/group of symptoms might help to elucidate further the role of inflammation in depression. Animal experimental studies suggest that inflammatory cytokines communicate with the brain using a number of pathways, and affect mood, cognition and behaviour by altering neurotransmitter metabolism, activating the hypothalamic–pituitary–adrenal (HPA) axis, increasing oxidative stress, and reducing synaptic plasticity possibly as a result of activation of microglia (a marker of neuroinflammation) (Raison *et al.* 2006; Dantzer *et al.* 2008). It remains to be seen whether neuroinflammation corresponds with severity of depression or response to antidepressant treatment in humans.

## Evidence for a role of inflammation, atopy and autoimmunity in psychosis

Epidemiological studies indicate a role of infection, inflammation and autoimmunity in schizophrenia (Khandaker *et al.* 2015; Khandaker & Dantzer, 2016). Schizophrenia is associated with increased prevalence of various infections, including neurotropic viruses from the *Herpesviridae* family, and the intracellular parasite, *Toxoplasma gondii*. However, it is unclear whether increased infection is a result of poor lifestyle, so more convincing results have come from longitudinal studies. Exposure to a variety of infection during foetal development, including herpes simplex virus type 2 (HSV-2), cytomegalovirus (CMV), influenza, *T. gondii*, non-specific viral/bacterial infection and elevated maternal inflammatory marker levels, is associated with risk of schizophrenia and related psychotic illness in adult life, though the strength of association for each of these exposures vary; reviewed (Brown & Derkits, 2010; Khandaker *et al*. 2012, 2013). A linear, dose–response relationship has been reported between hospitalisation with a serious infection (CNS or non-CNS) during childhood and risk of adult psychotic disorders (Benros *et al.* 2011).

Similar to depression, antipsychotic naïve first episode psychosis and acute psychotic relapse are associated with increased serum levels of proinflammatory cytokines, such as IL-1*β*, IL-6, TNF-*α* and decreased serum levels of the anti-inflammatory cytokine, IL-10, which tend to normalise after remission of symptoms with antipsychotic treatment (Miller *et al.* 2011; Upthegrove *et al.* 2014; Goldsmith *et al.* 2016). Serum cytokine levels, including IL-6 are associated with illness severity, duration and anti-psychotic treatment (Maes *et al.* 1994; Miller *et al.* 2011). IL-1*β* and IL-6 levels have been reported to be elevated in the cerebrospinal fluid (CSF) of schizophrenia patients (Garver *et al.* 2003). However, it is not clear to what extent peripheral inflammation is related to inflammatory activity in the CNS, so further studies are needed. Recently population-based longitudinal studies have shown that elevated IL-6 and CRP levels in childhood or adolescence are associated with increased risk of developing psychotic symptoms (Khandaker *et al.* 2014*a*) and schizophrenia (Metcalf *et al.* 2017) subsequently in adulthood. As with depression, the longitudinal studies indicate that inflammation could be a causal risk factor for psychosis. Adverse experiences in early-life could be a source of low-grade systemic inflammation in healthy people, as levels of circulating inflammatory markers in adults are associated with childhood adversity/maltreatment (Baumeister *et al.* 2016) and sub-optimal foetal environment as indexed by low birth weight (Tzoulaki *et al.* 2008). Therefore, whether low-grade systemic inflammation mediates the relationship between early-life adversity and adult depression or psychosis is a hypothesis that needs testing.

Further support for a role of the immune system in schizophrenia comes from studies pointing to links with atopy and autoimmunity (Benros *et al.* 2011). There is some evidence that childhood atopic disorders such as asthma, eczema are associated with increased risk of psychotic symptoms in adolescence (Khandaker *et al.* 2014*b*) and schizophrenia in adulthood (Pedersen *et al.* 2012). The prevalence of autoimmune conditions is increased in people with schizophrenia and their unaffected first-degree relatives (Eaton *et al.* 2006). Schizophrenia is associated with serum antibodies against dietary antigens, such as gliadin and casein (Lachance & McKenzie, 2014). Increased autoantibodies against neuronal cell surface targets, such as N-methyl-D-aspartate receptor (NMDAR) and components of the voltage-gated potassium channel complex, have been reported in some cases of psychosis in some (Zandi *et al.* 2011; Steiner *et al.* 2013), but not all studies (de Witte *et al.* 2015); reviewed (Pollak *et al.* 2014). Anti-NMDAR antibodies have been typically associated with the eponymous encephalitis, which is often associated with psychotic symptoms. Not all anti-NMADR antibody positive cases of psychosis have classic features of encephalitis, suggesting these antibodies might be a cause of psychosis in some people. Similarly, elimination of autoantibodies by plasmapheresis improves psychotic symptoms in some cases of first episode psychosis (Zandi *et al*. 2011, 2014). However, anti-NMDAR antibodies are also increased in depression and bipolar disorder (Pearlman & Najjar, 2014). It remains to be established whether antibody positive patients differ from antibody-negative patients in terms of underlying pathology and response to antipsychotic treatment, and whether immunomodulatory treatments are effective in alleviating psychotic symptoms in this group using RCT. There is limited data on the relationships between neuronal autoantibodies and other aspects of immunity such as cytokines and microglial activation. Nevertheless, given these antibodies are present in about 5% of patients with first episode psychosis (Zandi *et al.* 2011), some psychiatric services in the UK now screen for these antibodies in new onset cases.

Neuroimaging and genome wide association studies (GWAS) support a role for the immune system in schizophrenia. Neuroimaging studies using PET provide evidence for neuroinflammation in patients with schizophrenia and in persons with subclinical symptoms, who are at high risk of psychosis and are related to at-risk symptom severity (Bloomfield *et al.* 2016). Evidence of microglial activation in the entire grey matter and hippocampus indicates that neuroinflammation might contribute to grey matter volume loss and cognitive deterioration in schizophrenia (Doorduin *et al.* 2009). The largest GWAS of schizophrenia to date has shown that in addition to genes expressed in the brain, schizophrenia is associated with genes involved in adaptive immunity (CD19 and CD 20 B-lymphocytes) and the major histocompatibility complex (MHC) region on chromosome six (Schizophrenia Working Group of the Psychiatric Genomics, 2014). The association between schizophrenia and the MHC locus arises in part from variations of the complement component 4 (*C4*) genes. C4 is a critical component of the classical complement cascade, an innate immune system pathway that recognizes and eliminates pathogens and cellular debris. The brain expression of *C4A* is increased in schizophrenia (Sekar *et al.* 2016). In mice, C4 tags synapses to be eliminated by microglial cells (synaptic pruning) that express C4 receptors during postnatal development. Excessive complement activity could lead to altered neurodevelopment as a result of excessive synaptic pruning.

## Inflammation and response to psychotropic treatments

Inflammation is associated with poor response to antidepressant and antipsychotic treatments. About a third of depressed patients have elevated circulating CRP levels (Wium-Andersen *et al.* 2013) while a third of patients are antidepressant resistant (Nemeroff, 2007) – this may not be a coincidence. Indeed, activation of the inflammatory system as reflected by elevated serum concentrations of IL-6 and other cytokines predicts poor response to antidepressants (O'Brien *et al.* 2007; Carvalho *et al.* 2013). Patients who do not respond to selective serotonin reuptake inhibitors and other antidepressants continue to show elevated levels of IL-6, CRP and other inflammatory markers (Maes *et al.* 1997). Findings from patients with psychosis are similar. Patients with first episode psychosis who do not respond to antipsychotic drugs have elevated levels of IL-6 and INF-*γ* at the start of treatment compared with responders (Mondelli *et al.* 2015). Furthermore, non-responders continue to show elevated levels of these inflammatory markers after 3 months of antipsychotic treatment compared with responders (Mondelli *et al.* 2015). While these findings are illuminating, longitudinal studies of inflammation and treatment response based on incident cases are limited. Inflammatory marker levels can be influenced by psychological stress, body mass, life style (e.g. smoking, alcohol use) and some psychotropic drugs. So further research is needed to understand whether and how measuring inflammation in clinical setting could be useful for predicting response to antidepressant/antipsychotic treatment, and for identifying patients who are likely to benefit from immunomodulatory treatments.

It is thought that depressed patients with elevated inflammatory markers display resistance to antidepressants because these drugs do not rectify inflammation-related pathologies such as activation of the kynurenine pathway (Christmas *et al.* 2011). Inflammatory cytokines such as IFN and IL-6 activate indoleamine 2,3-dioxygenase (IDO), an enzyme that shifts metabolism of tryptophan towards kynurenine (Haroon *et al.* 2012). Further metabolism of kynurenine in the brain leads to increased production of neurotoxic and potentially depressogenic metabolites such as 3-hydroxykynurenine (3-HK) and quinolinic acid. In mice, lipopolysaccharide-induced systemic inflammation leads to depression-like behaviour through activation of IDO (O'Connor *et al.* 2009). Blocking inflammation with minocycline (a broad spectrum antibiotic) (O'Connor *et al.* 2009) or an anti-IL-6 monoclonal antibody (Hodes *et al.* 2014) prevents development of depression-like behaviour. Pertinent to psychosis, kynurenic acid is the only naturally occurring NMDAR antagonist in the human CNS (Schwarcz & Pellicciari, 2002). NMDAR antagonism and glutamatergic hypofunction have long been proposed to underlie psychotic symptoms and cognitive dysfunction in schizophrenia (Carlsson & Carlsson, 1990). Indeed, there is evidence that levels of kynurenine and kynurenic acid are elevated in the CSF and brain tissue of schizophrenia patients compared with healthy controls (Erhardt *et al.* 2001; Schwarcz *et al.* 2001). In future, examination of markers of inflammation and IDO activation in peripheral blood and in CSF in different stages of illness, their relationship with treatment response would be helpful to understand fully the implications of these findings for depression and psychosis.

## Effectiveness of immunomodulatory treatment in depression and psychosis

Anti-inflammatory drugs may be helpful for patients with depression and psychosis. A meta-analysis of RCTs of non-steroidal anti-inflammatory drugs (NSAIDs), administered as sole treatment or as adjunct to antidepressants, indicates that they are more effective than placebo in treating depression (Kohler *et al.* 2014). The effect size for Celecoxib (a selective cyclooxygenase 2 inhibitor) based on 10 RCTs was SMD = −0.29 (95% CI −0.49 to −0.08). However, cyclooxygenase-2 inhibitors increase risk of cardiovascular disease (Mukherjee *et al.* 2001), a known comorbidity for depression, so their use in this patient group may be problematic. Besides, it unclear whether improvements in mood is due to improvements in physical illness or due to the effect of NSAIDs on pathways other than inflammation such as glucocorticoid receptor (Nikkheslat *et al.* 2015).

More recently studies have examined the effectiveness of cytokine modulators (e.g. anti-cytokine monoclonal antibodies and cytokine inhibitors) for depression. We have conducted a systematic review and meta-analysis of secondary outcome data on depressive symptoms from clinical trials of cytokine modulators in patients with chronic inflammatory illnesses. Based on seven RCTs, we have found that cytokine modulators have a significant antidepressant effect; effect size, SMD = 0.40 (95% CI 0.22–0.59) (Kappelmann *et al.* 2016). Crucially, improvements in depressive symptoms are independent of improvements in physical illness, suggesting mood improvement is not merely a by-product of physical improvement; cytokines may indeed be causally related to depressive symptoms. Only one RCT to date has investigated the effectiveness of a monoclonal antibody using depression as a primary outcome (Raison *et al.* 2013). Although overall no benefit of infliximab (anti-TNF monoclonal antibody) was observed, the study found that the higher the level of inflammation at the start of trial the greater the improvement in depressive symptoms by the end of trial (Raison *et al.* 2013). Together, these findings indicate that cytokine modulating drugs could be useful for depressed patients, particularly those with evidence of inflammation, but further work is needed to establish their efficacy, safety and cost-benefit for treating depression.

In schizophrenia, a meta-analysis of RCTs of NSAIDs as adjuncts to antipsychotics has shown promising results especially for aspirin and non-acetyl cysteine (Sommer *et al.* 2014). Dopaminergic drugs, which include all anti-psychotics currently in use, are effective in controlling positive symptoms, but cognitive dysfunction and negative symptoms rarely respond to these drugs. This may indicate fundamental differences in the mechanism underlying different types of symptoms. Preclinical studies of peripheral immune-to-brain communication lend support for a role of inflammation in the pathogenesis of these difficult-to-treat symptoms (Khandaker *et al*. 2015; Khandaker & Dantzer, 2016). In future, targeting these symptoms with immunological treatment could be a clinically fruitful strategy. Indeed, minocycline, a centrally acting tetracyclic anti-inflammatory agent, improves negative symptoms and cognitive function in schizophrenia (Chaudhry *et al.* 2012). Celecoxib may improve cognitive function in early stages of schizophrenia (Muller *et al.* 2002). Recently, a small, proof-of-concept, open-label study of tocilizumab (anti-IL-6 receptor monoclonal antibody) as adjunct to antipsychotic treatment showed improvements in cognitive function in schizophrenia (Miller *et al.* 2016), but more definitive trials are needed. A subgroup of patients with depression (Wium-Andersen *et al.* 2013) and psychosis (Mondelli *et al.* 2015) show evidence of inflammation, so RCTs of anti-inflammatory drugs based on ‘inflamed’ rather than all patients of depression/psychosis are likely to be useful.

## Transdiagnostic effect of inflammation and the common cause hypothesis

The association between inflammation and psychiatric disorders transcends traditional diagnostic boundaries. In addition to depression and psychosis, evidence for a link with inflammation exists for a number of disorders, including anxiety (Rossi *et al.* 2012; Khandaker *et al.* 2016), post-traumatic stress disorder (Eraly *et al.* 2014), autism (Brown *et al.* 2014) and dementias (Schmidt *et al.* 2002). Inflammation is likely to be relevant for some, *not all*, cases of depression/psychosis, because, as with most chronic illnesses, no risk factor alone is necessary or sufficient for causing an illness. Inflammation is no different in this regard; evidence for inflammation is seen in a sup-group of patients with depression and psychosis (Wium-Andersen *et al.* 2013; Mondelli *et al.* 2015). One explanation for the transdiagnostic effect could be that inflammation contributes to features common to different psychiatric syndromes such as fatigue, anhedonia and cognitive difficulties. Furthermore, this lack of specificity is not unique to inflammation. It is well known that there is overlap in the genetic risk for depression, schizophrenia and bipolar disorder. Psychological stress is a common risk factor for many physical and psychiatric illnesses.

Taking a broader perspective on causal models for illness, the idea of *specificity of association* between a risk factor and a disease fits well causal models for some illnesses (e.g. Koch's postulates and Tuberculosis), but might be problematic in the case of chronic illnesses. Smoking is associated with numerous illnesses, including cancers and cardiovascular diseases. Similarly, inflammation might be a common underlying mechanism for a number of chronic illnesses of adult life such as depression, schizophrenia, coronary heart disease and diabetes mellitus that are highly comorbid with each other. All of these illnesses are associated with inflammation (Pradhan *et al.* 2001; Danesh *et al.* 2004; Khandaker *et al.* 2014*a*; Metcalf *et al.* 2017), which could be linked with early-life factors influencing inflammatory regulation, such as impaired foetal development or childhood maltreatment. This idea is consistent with the common-cause or developmental programming hypothesis by David Barker (Barker *et al.* 1993*a*). Developmental programming refers to permanent alteration in physiological system(s) following exposure to adversity during a specific ‘developmental window’. The resulting physiological alterations, in combination with genetic and/or other environmental factors, can increase risks of several diseases in adulthood. Empirical evidence from longitudinal studies supports the developmental programming hypothesis. Low birth weight (a marker of suboptimal foetal development) and childhood maltreatment are associated with increased circulating CRP levels (Tzoulaki *et al.* 2008) as well as risks of heart disease, diabetes mellitus, depression and schizophrenia (Barker *et al.* 1993*b*; Abel *et al.* 2010). Indeed, there is evidence that inflammation moderates the association between childhood maltreatment and risk of depression in adulthood (Danese *et al.* 2008). In future, population-based epidemiological, clinical and experimental studies are needed to understand whether and how inflammation contributes to the comorbidity between chronic cardio-metabolic and psychiatric illnesses of adult life. Experimental studies would be particularly useful to understand how early-life adversity might ‘programme’ the immune system.

## Conclusions

Pathophysiologic explanations and pharmacotherapy for major psychiatric disorders such as depression and schizophrenia are predicated on monoamine neurotransmitters. However, heterogeneity in clinical presentation, illness course and treatment response suggest other mechanisms are involved. A better understanding of the role of immune system could be paradigm changing for psychiatry by providing novel mechanisms and treatments for these disorders, which are among leading causes of disability worldwide. The field now needs studies to investigate causality of association and RCTs of novel immunotherapies for depression and psychosis. In future, immunotherapeutic strategies could include immune-phenotyping of patients to predict treatment response and anti-inflammatory treatment for patients with evidence of inflammation. Shifting the focus of research from syndrome to symptom (or constellation of symptoms) may help to fully elucidate the role of immune system in the aetiology of psychiatric disorders. Accumulating evidence from preclinical studies suggests inflammation is a key mechanism underlying cognition and behaviour, which needs to be translated into human studies of affective and cognitive neuroscience. A clearer understanding of the immunological aspects of psychiatric disorders may lead to novel approaches to diagnosis and treatment but would require close collaborations between clinicians and researchers to deliver high-quality translational research.

Immunopsychiatry: Key facts

• Depression is common in people with a chronic inflammatory illness, and immuno-activation leads to depressive symptoms in patients and healthy volunteers
• Schizophrenia and related psychotic disorders are associated with prenatal and childhood infections, atopic disorders and autoimmunity
• Elevated circulating inflammatory markers increase future risk of depression and psychosis in healthy people suggesting inflammation might cause these disorders
• Circulating inflammatory markers are elevated in acute depression and psychosis, which tend to normalise after recovery, but continue to be elevated in treatment resistant cases
• There is evidence of neuroinflammation in schizophrenia as demonstrated by activation of microglia in patients as well as those who are at risk of developing the illness
• Anti-NMDA receptor and anti-VGKC antibodies may be responsible for about 5% of all cases of psychosis
• Elevated circulating inflammatory markers are associated with poor response to antidepressant and antipsychotic treatment
• Measuring circulating inflammatory markers may aid treatment decisions
• Emerging evidence indicates anti-inflammatory agents may be effective treatments for patients with depression and psychosis particularly those with evidence of inflammation
• Inflammation might be a common mechanism underlying the comorbidities between depression, schizophrenia, coronary heart disease and diabetes mellitus

## References

[ref1] Abel KM, Wicks S, Susser ES, Dalman C, Pedersen MG, Mortensen PB, Webb RT (2010). Birth weight, schizophrenia, and adult mental disorder: is risk confined to the smallest babies? Archives of General Psychiatry 67, 923–930. 20819986 10.1001/archgenpsychiatry.2010.100

[ref2] Barker DJ, Gluckman PD, Godfrey KM, Harding JE, Owens JA, Robinson JS (1993 *a*). Fetal nutrition and cardiovascular disease in adult life. Lancet 341, 938–941. 8096277 10.1016/0140-6736(93)91224-a

[ref3] Barker DJ, Hales CN, Fall CH, Osmond C, Phipps K, Clark PM (1993 *b*). Type 2 (non-insulin-dependent) diabetes mellitus, hypertension and hyperlipidaemia (syndrome X): relation to reduced fetal growth. Diabetologia 36, 62–67. 8436255 10.1007/BF00399095

[ref4] Baumeister D, Akhtar R, Ciufolini S, Pariante CM, Mondelli V (2016). Childhood trauma and adulthood inflammation: a meta-analysis of peripheral C-reactive protein, interleukin-6 and tumour necrosis factor-alpha. Molecular Psychiatry 21, 642–649. 26033244 10.1038/mp.2015.67 PMC4564950

[ref5] Benros ME, Nielsen PR, Nordentoft M, Eaton WW, Dalton SO, Mortensen PB (2011). Autoimmune diseases and severe infections as risk factors for schizophrenia: a 30-year population-based register study. American Journal of Psychiatry 168, 1303–1310. 22193673 10.1176/appi.ajp.2011.11030516

[ref6] Bloomfield PS, Selvaraj S, Veronese M, Rizzo G, Bertoldo A, Owen DR, Bloomfield MA, Bonoldi I, Kalk N, Turkheimer F, McGuire P, de Paola V, Howes OD (2016). Microglial activity in people at ultra high risk of psychosis and in schizophrenia: an [(11)C]PBR28 PET brain imaging study. American Journal of Psychiatry 173, 44–52. 26472628 10.1176/appi.ajp.2015.14101358 PMC4821370

[ref7] Bonaccorso S, Marino V, Biondi M, Grimaldi F, Ippoliti F, Maes M (2002). Depression induced by treatment with interferon-alpha in patients affected by hepatitis C virus. Journal of Affective Disorders 72, 237–241. 12450640 10.1016/s0165-0327(02)00264-1

[ref8] Brown AS, Derkits EJ (2010). Prenatal infection and schizophrenia: a review of epidemiologic and translational studies. American Journal of Psychiatry 167, 261–280. 20123911 10.1176/appi.ajp.2009.09030361 PMC3652286

[ref9] Brown AS, Sourander A, Hinkka-Yli-Salomaki S, McKeague IW, Sundvall J, Surcel HM (2014). Elevated maternal C-reactive protein and autism in a national birth cohort. Molecular Psychiatry 19, 259–264. 23337946 10.1038/mp.2012.197 PMC3633612

[ref10] Capuron L, Gumnick JF, Musselman DL, Lawson DH, Reemsnyder A, Nemeroff CB, Miller AH (2002). Neurobehavioral effects of interferon-alpha in cancer patients: phenomenology and paroxetine responsiveness of symptom dimensions. Neuropsychopharmacology 26, 643–652. 11927189 10.1016/S0893-133X(01)00407-9

[ref11] Carlsson M, Carlsson A (1990). Schizophrenia: a subcortical neurotransmitter imbalance syndrome? Schizophrenia Bulletin 16, 425–432. 1981107 10.1093/schbul/16.3.425

[ref12] Carvalho LA, Torre JP, Papadopoulos AS, Poon L, Juruena MF, Markopoulou K, Cleare AJ, Pariante CM (2013). Lack of clinical therapeutic benefit of antidepressants is associated overall activation of the inflammatory system. Journal of Affective Disorders 148, 136–140. 23200297 10.1016/j.jad.2012.10.036

[ref13] Chaudhry IB, Hallak J, Husain N, Minhas F, Stirling J, Richardson P, Dursun S, Dunn G, Deakin B (2012). Minocycline benefits negative symptoms in early schizophrenia: a randomised double-blind placebo-controlled clinical trial in patients on standard treatment. Journal of Psychopharmacology 26, 1185–1193. 22526685 10.1177/0269881112444941

[ref14] Christmas DM, Potokar J, Davies SJ (2011). A biological pathway linking inflammation and depression: activation of indoleamine 2,3-dioxygenase. Neuropsychiatric Disease and Treatment 7, 431–439. 21792309 10.2147/NDT.S17573 PMC3140295

[ref15] Danese A, Moffitt TE, Pariante CM, Ambler A, Poulton R, Caspi A (2008). Elevated inflammation levels in depressed adults with a history of childhood maltreatment. Archives of General Psychiatry 65, 409–415. 18391129 10.1001/archpsyc.65.4.409 PMC2923056

[ref16] Danesh J, Wheeler JG, Hirschfield GM, Eda S, Eiriksdottir G, Rumley A, Lowe GD, Pepys MB, Gudnason V (2004). C-reactive protein and other circulating markers of inflammation in the prediction of coronary heart disease. New England Journal of Medicine 350, 1387–1397. 15070788 10.1056/NEJMoa032804

[ref17] Dantzer R, O'Connor JC, Freund GG, Johnson RW, Kelley KW (2008). From inflammation to sickness and depression: when the immune system subjugates the brain. Nature Reviews Neuroscience 9, 46–56. 18073775 10.1038/nrn2297 PMC2919277

[ref18] de Witte LD, Hoffmann C, van Mierlo HC, Titulaer MJ, Kahn RS, Martinez-Martinez P, D European Consortium of Autoimmune Mental (2015). Absence of N-Methyl-D-aspartate receptor IgG autoantibodies in schizophrenia: the importance of cross-validation studies. JAMA Psychiatry 72, 731–733. 25970159 10.1001/jamapsychiatry.2015.0526

[ref19] Dickens C, Creed F (2001). The burden of depression in patients with rheumatoid arthritis. Rheumatology *(*Oxford*)* 40, 1327–1330. 11752500 10.1093/rheumatology/40.12.1327

[ref20] Doorduin J, de Vries EF, Willemsen AT, de Groot JC, Dierckx RA, Klein HC (2009). Neuroinflammation in schizophrenia-related psychosis: a PET study. Journal of Nuclear Medicine 50, 1801–1807. 19837763 10.2967/jnumed.109.066647

[ref21] Eaton WW, Byrne M, Ewald H, Mors O, Chen CY, Agerbo E, Mortensen PB (2006). Association of schizophrenia and autoimmune diseases: linkage of Danish national registers. American Journal of Psychiatry 163, 521–528. 16513876 10.1176/appi.ajp.163.3.521

[ref22] Eraly SA, Nievergelt CM, Maihofer AX, Barkauskas DA, Biswas N, Agorastos A, O'Connor DT, Baker DG, T Marine Resiliency Study (2014). Assessment of plasma C-reactive protein as a biomarker of posttraumatic stress disorder risk. JAMA Psychiatry 71, 423–431. 24576974 10.1001/jamapsychiatry.2013.4374 PMC4032578

[ref23] Erhardt S, Blennow K, Nordin C, Skogh E, Lindstrom LH, Engberg G (2001). Kynurenic acid levels are elevated in the cerebrospinal fluid of patients with schizophrenia. Neuroscience Letters 313, 96–98. 11684348 10.1016/s0304-3940(01)02242-x

[ref24] Garver DL, Tamas RL, Holcomb JA (2003). Elevated interleukin-6 in the cerebrospinal fluid of a previously delineated schizophrenia subtype. Neuropsychopharmacology 28, 1515–1520. 12799618 10.1038/sj.npp.1300217

[ref25] Gimeno D, Kivimaki M, Brunner EJ, Elovainio M, De Vogli R, Steptoe A, Kumari M, Lowe GD, Rumley A, Marmot MG, Ferrie JE (2009). Associations of C-reactive protein and interleukin-6 with cognitive symptoms of depression: 12-year follow-up of the Whitehall II study. Psychological Medicine 39, 413–423. 18533059 10.1017/S0033291708003723 PMC2788760

[ref26] Goldsmith DR, Rapaport MH, Miller BJ (2016). A meta-analysis of blood cytokine network alterations in psychiatric patients: comparisons between schizophrenia, bipolar disorder and depression. Molecular Psychiatry 21, 1696–1709. 26903267 10.1038/mp.2016.3 PMC6056174

[ref27] Haapakoski R, Mathieu J, Ebmeier KP, Alenius H, Kivimaki M (2015). Cumulative meta-analysis of interleukins 6 and 1beta, tumour necrosis factor alpha and C-reactive protein in patients with major depressive disorder. Brain Behaviour and Immunity 49, 206–215. 10.1016/j.bbi.2015.06.001 PMC4566946 26065825

[ref28] Haroon E, Raison CL, Miller AH (2012). Psychoneuroimmunology meets neuropsychopharmacology: translational implications of the impact of inflammation on behavior. Neuropsychopharmacology 37, 137–162. 21918508 10.1038/npp.2011.205 PMC3238082

[ref29] Harrison NA, Brydon L, Walker C, Gray MA, Steptoe A, Critchley HD (2009). Inflammation causes mood changes through alterations in subgenual cingulate activity and mesolimbic connectivity. Biological Psychiatry 66, 407–414. 19423079 10.1016/j.biopsych.2009.03.015 PMC2885494

[ref30] Hodes GE, Pfau ML, Leboeuf M, Golden SA, Christoffel DJ, Bregman D, Rebusi N, Heshmati M, Aleyasin H, Warren BL, Lebonte B, Horn S, Lapidus KA, Stelzhammer V, Wong EH, Bahn S, Krishnan V, Bolanos-Guzman CA, Murrough JW, Merad M, Russo SJ (2014). Individual differences in the peripheral immune system promote resilience versus susceptibility to social stress. Proceedings of the National Academy of Sciences of the United States of America 111, 16136–16141. 25331895 10.1073/pnas.1415191111 PMC4234602

[ref31] Howren MB, Lamkin DM, Suls J (2009). Associations of depression with C-reactive protein, IL-1, and IL-6: a meta-analysis. Psychosomatic Medicine 71, 171–186. 19188531 10.1097/PSY.0b013e3181907c1b

[ref32] Jokela M, Virtanen M, Batty GD, Kivimaki M (2016). Inflammation and specific symptoms of depression. JAMA Psychiatry 73, 87–88. 26579988 10.1001/jamapsychiatry.2015.1977

[ref33] Kappelmann N, Lewis G, Dantzer R, Jones PB, Khandaker GM (2016). Antidepressant activity of anti-cytokine treatment: a systematic review and meta-analysis of clinical trials of chronic inflammatory conditions. Molecular Psychiatry. doi: 10.1038/mp.2016.167. [Epub ahead of print]. PMC5794896 27752078

[ref34] Khandaker GM, Cousins L, Deakin J, Lennox BR, Yolken R, Jones PB (2015). Inflammation and immunity in schizophrenia: implications for pathophysiology and treatment. The Lancet Psychiatry 2, 258–270. 26359903 10.1016/S2215-0366(14)00122-9 PMC4595998

[ref35] Khandaker GM, Dantzer R (2016). Is there a role for immune-to-brain communication in schizophrenia? Psychopharmacology *(*Berlin*)* 233, 1559–1573. 26037944 10.1007/s00213-015-3975-1 PMC4671307

[ref36] Khandaker GM, Pearson RM, Zammit S, Lewis G, Jones PB (2014 *a*). Association of serum interleukin 6 and C-reactive protein in childhood with depression and psychosis in young adult life: a population-based longitudinal study. JAMA Psychiatry 71, 1121–1128. 25133871 10.1001/jamapsychiatry.2014.1332 PMC4561502

[ref37] Khandaker GM, Zammit S, Lewis G, Jones PB (2014 *b*). A population-based study of atopic disorders and inflammatory markers in childhood before psychotic experiences in adolescence. Schizophrenia Research 152, 139–145. 24268471 10.1016/j.schres.2013.09.021 PMC3906534

[ref38] Khandaker GM, Zammit S, Lewis G, Jones PB (2016). Association between serum C-reactive protein and DSM-IV generalized anxiety disorder in adolescence: findings from the ALSPAC cohort. Neurobiology of Stress 4, 55–61. 27830164 10.1016/j.ynstr.2016.02.003 PMC5098599

[ref39] Khandaker GM, Zimbron J, Dalman C, Lewis G, Jones PB (2012). Childhood infection and adult schizophrenia: a meta-analysis of population-based studies. Schizophrenia Research 139, 161–168. 22704639 10.1016/j.schres.2012.05.023 PMC3485564

[ref40] Khandaker GM, Zimbron J, Lewis G, Jones PB (2013). Prenatal maternal infection, neurodevelopment and adult schizophrenia: a systematic review of population-based studies. Psychological Medicine 43, 239–257. 22717193 10.1017/S0033291712000736 PMC3479084

[ref41] Kohler O, Benros ME, Nordentoft M, Farkouh ME, Iyengar RL, Mors O, Krogh J (2014). Effect of anti-inflammatory treatment on depression, depressive symptoms, and adverse effects: a systematic review and meta-analysis of randomized clinical trials. JAMA Psychiatry 71, 1381–1391. 25322082 10.1001/jamapsychiatry.2014.1611

[ref42] Lachance LR, McKenzie K (2014). Biomarkers of gluten sensitivity in patients with non-affective psychosis: a meta-analysis. Schizophrenia Research 152, 521–527. 24368154 10.1016/j.schres.2013.12.001

[ref43] Maes M, Bosmans E, De Jongh R, Kenis G, Vandoolaeghe E, Neels H (1997). Increased serum IL-6 and IL-1 receptor antagonist concentrations in major depression and treatment resistant depression. Cytokine 9, 853–858. 9367546 10.1006/cyto.1997.0238

[ref44] Maes M, Meltzer HY, Bosmans E (1994). Immune-inflammatory markers in schizophrenia: comparison to normal controls and effects of clozapine. Acta Psychiatrica Scandinavica 89, 346–351. 8067274 10.1111/j.1600-0447.1994.tb01527.x

[ref45] Metcalf SA, Jones PB, Nordstrom T, Timonen M, Maki P, Miettunen J, Jaaskelainen E, Jarvelin MR, Stochl J, Murray GK, Veijola J, Khandaker GM (2017). Serum C-reactive protein in adolescence and risk of schizophrenia in adulthood: a prospective birth cohort study. Brain Behaviour and Immunity 59, 253–259. 10.1016/j.bbi.2016.09.008 PMC5176002 27622678

[ref46] Miller BJ, Buckley P, Seabolt W, Mellor A, Kirkpatrick B (2011). Meta-analysis of cytokine alterations in schizophrenia: clinical status and antipsychotic effects. Biological Psychiatry 70, 663–671. 21641581 10.1016/j.biopsych.2011.04.013 PMC4071300

[ref47] Miller BJ, Dias JK, Lemos HP, Buckley PF (2016). An open-label, pilot trial of adjunctive tocilizumab in schizophrenia. Journal of Clinical Psychiatry 77, 275–276. 26930525 10.4088/JCP.15l09920

[ref48] Mondelli V, Ciufolini S, Belvederi Murri M, Bonaccorso S, Di Forti M, Giordano A, Marques TR, Zunszain PA, Morgan C, Murray RM, Pariante CM, Dazzan P (2015). Cortisol and inflammatory biomarkers predict poor treatment response in first episode psychosis. Schizophrenia Bulletin 41, 1162–1170. 25829375 10.1093/schbul/sbv028 PMC4535637

[ref49] Mukherjee D, Nissen SE, Topol EJ (2001). Risk of cardiovascular events associated with selective COX-2 inhibitors. JAMA 286, 954–959. 11509060 10.1001/jama.286.8.954

[ref50] Muller N, Riedel M, Scheppach C, Brandstatter B, Sokullu S, Krampe K, Ulmschneider M, Engel RR, Moller HJ, Schwarz MJ (2002). Beneficial antipsychotic effects of celecoxib add-on therapy compared to risperidone alone in schizophrenia. American Journal of Psychiatry 159, 1029–1034. 12042193 10.1176/appi.ajp.159.6.1029

[ref51] Nemeroff CB (2007). Prevalence and management of treatment-resistant depression. Journal of Clinical Psychiatry 68(Suppl 8), 17–25. 17640154

[ref52] Nikkheslat N, Zunszain PA, Horowitz MA, Barbosa IG, Parker JA, Myint AM, Schwarz MJ, Tylee AT, Carvalho LA, Pariante CM (2015). Insufficient glucocorticoid signaling and elevated inflammation in coronary heart disease patients with comorbid depression. Brain Behaviour and Immunity 48, 8–18. 10.1016/j.bbi.2015.02.002 25683698

[ref53] O'Brien SM, Scully P, Fitzgerald P, Scott LV, Dinan TG (2007). Plasma cytokine profiles in depressed patients who fail to respond to selective serotonin reuptake inhibitor therapy. Journal of Psychiatric Research 41, 326–331. 16870211 10.1016/j.jpsychires.2006.05.013

[ref54] O'Connor JC, Lawson MA, Andre C, Moreau M, Lestage J, Castanon N, Kelley KW, Dantzer R (2009). Lipopolysaccharide-induced depressive-like behavior is mediated by indoleamine 2,3-dioxygenase activation in mice. Molecular Psychiatry 14, 511–522. 18195714 10.1038/sj.mp.4002148 PMC2683474

[ref55] Pearlman DM, Najjar S (2014). Meta-analysis of the association between N-methyl-d-aspartate receptor antibodies and schizophrenia, schizoaffective disorder, bipolar disorder, and major depressive disorder. Schizophrenia Research 157, 249–258. 24882425 10.1016/j.schres.2014.05.001

[ref56] Pedersen MS, Benros ME, Agerbo E, Borglum AD, Mortensen PB (2012). Schizophrenia in patients with atopic disorders with particular emphasis on asthma: a Danish population-based study. Schizophrenia Research 138, 58–62. 22391212 10.1016/j.schres.2012.02.019

[ref57] Pollak TA, McCormack R, Peakman M, Nicholson TR, David AS (2014). Prevalence of anti-N-methyl-D-aspartate (NMDA) receptor [corrected] antibodies in patients with schizophrenia and related psychoses: a systematic review and meta-analysis. Psychological Medicine 44, 2475–2487. 24330811 10.1017/S003329171300295X

[ref58] Pradhan AD, Manson JE, Rifai N, Buring JE, Ridker PM (2001). C-reactive protein, interleukin 6, and risk of developing type 2 diabetes mellitus. JAMA 286, 327–334. 11466099 10.1001/jama.286.3.327

[ref59] Raison CL, Capuron L, Miller AH (2006). Cytokines sing the blues: inflammation and the pathogenesis of depression. Trends in Immunology 27, 24–31. 16316783 10.1016/j.it.2005.11.006 PMC3392963

[ref60] Raison CL, Rutherford RE, Woolwine BJ, Shuo C, Schettler P, Drake DF, Haroon E, Miller AH (2013). A randomized controlled trial of the tumor necrosis factor antagonist infliximab for treatment-resistant depression: the role of baseline inflammatory biomarkers. JAMA Psychiatry 70, 31–41. 22945416 10.1001/2013.jamapsychiatry.4 PMC4015348

[ref61] Rossi S, Sacchetti L, Napolitano F, De Chiara V, Motta C, Studer V, Musella A, Barbieri F, Bari M, Bernardi G, Maccarrone M, Usiello A, Centonze D (2012). Interleukin-1beta causes anxiety by interacting with the endocannabinoid system. Journal of Neuroscience 32, 13896–13905. 23035099 10.1523/JNEUROSCI.1515-12.2012 PMC6704788

[ref62] Schizophrenia Working Group of the Psychiatric Genomics (2014). Biological insights from 108 schizophrenia-associated genetic loci. Nature 511, 421–427. 25056061 10.1038/nature13595 PMC4112379

[ref63] Schmidt R, Schmidt H, Curb JD, Masaki K, White LR, Launer LJ (2002). Early inflammation and dementia: a 25-year follow-up of the Honolulu-Asia Aging Study. Annals of Neurology 52, 168–174. 12210786 10.1002/ana.10265

[ref64] Schwarcz R, Pellicciari R (2002). Manipulation of brain kynurenines: glial targets, neuronal effects, and clinical opportunities. Journal of Pharmacology and Experimental Therapeutics 303, 1–10. 12235226 10.1124/jpet.102.034439

[ref65] Schwarcz R, Rassoulpour A, Wu HQ, Medoff D, Tamminga CA, Roberts RC (2001). Increased cortical kynurenate content in schizophrenia. Biological Psychiatry 50, 521–530. 11600105 10.1016/s0006-3223(01)01078-2

[ref66] Sekar A, Bialas AR, de Rivera H, Davis A, Hammond TR, Kamitaki N, Tooley K, Presumey J, Baum M, Van Doren V, Genovese G, Rose SA, Handsaker RE, Daly MJ, Carroll MC, Stevens B, McCarroll SA, C Schizophrenia Working Group of the Psychiatric Genomics (2016). Schizophrenia risk from complex variation of complement component 4. Nature 530, 177–183. 26814963 10.1038/nature16549 PMC4752392

[ref67] Sommer IE, van Westrhenen R, Begemann MJ, de Witte LD, Leucht S, Kahn RS (2014). Efficacy of anti-inflammatory agents to improve symptoms in patients with schizophrenia: an update. Schizophrenia Bulletin 40, 181–191. 24106335 10.1093/schbul/sbt139 PMC3885306

[ref69] Steiner J, Walter M, Glanz W, Sarnyai Z, Bernstein HG, Vielhaber S, Kastner A, Skalej M, Jordan W, Schiltz K, Klingbeil C, Wandinger KP, Bogerts B, Stoecker W (2013). Increased prevalence of diverse N-methyl-D-aspartate glutamate receptor antibodies in patients with an initial diagnosis of schizophrenia: specific relevance of IgG NR1a antibodies for distinction from N-methyl-D-aspartate glutamate receptor encephalitis. JAMA Psychiatry 70, 271–278. 23344076 10.1001/2013.jamapsychiatry.86

[ref70] Tzoulaki I, Jarvelin MR, Hartikainen AL, Leinonen M, Pouta A, Paldanius M, Ruokonen A, Canoy D, Sovio U, Saikku P, Elliott P (2008). Size at birth, weight gain over the life course, and low-grade inflammation in young adulthood: Northern Finland 1966 Birth Cohort study. European Heart Journal 29, 1049–1056. 18403494 10.1093/eurheartj/ehn105

[ref71] Upthegrove R, Manzanares-Teson N, Barnes NM (2014). Cytokine function in medication-naive first episode psychosis: a systematic review and meta-analysis. Schizophrenia Research 155, 101–108. 24704219 10.1016/j.schres.2014.03.005

[ref72] Wium-Andersen MK, Orsted DD, Nielsen SF, Nordestgaard BG (2013). Elevated C-reactive protein levels, psychological distress, and depression in 73, 131 individuals. JAMA Psychiatry 70, 176–184. 23266538 10.1001/2013.jamapsychiatry.102

[ref73] Zalli A, Jovanova O, Hoogendijk WJ, Tiemeier H, Carvalho LA (2016). Low-grade inflammation predicts persistence of depressive symptoms. Psychopharmacology *(*Berlin*)* 233, 1669–1678. 25877654 10.1007/s00213-015-3919-9 PMC4828485

[ref74] Zandi MS, Deakin JB, Morris K, Buckley C, Jacobson L, Scoriels L, Cox AL, Coles AJ, Jones PB, Vincent A, Lennox BR (2014). Immunotherapy for patients with acute psychosis and serum N-methyl d-aspartate receptor (NMDAR) antibodies: a description of a treated case series. Schizophrenia Research 160, 193–195. 25468187 10.1016/j.schres.2014.11.001

[ref75] Zandi MS, Irani SR, Lang B, Waters P, Jones PB, McKenna P, Coles AJ, Vincent A, Lennox BR (2011). Disease-relevant autoantibodies in first episode schizophrenia. Journal of Neurology 258, 686–688. 20972895 10.1007/s00415-010-5788-9 PMC3065649

